# Major Surface Antigens in Zoonotic *Babesia*

**DOI:** 10.3390/pathogens11010099

**Published:** 2022-01-15

**Authors:** Stephane Delbecq

**Affiliations:** Centre de Biologie Structurale, Faculté de Pharmacie, University of Montpellier, UMR CNRS 5048, 34090 Montpellier, France; stephane.delbecq@umontpellier.fr

**Keywords:** glycosylphosphatidylinositol, protein structure, antigen

## Abstract

Human babesiosis results from a combination of tick tropism for humans, susceptibility of a host to sustain *Babesia* development, and contact with infected ticks. Climate modifications and increasing diagnostics have led to an expanded number of *Babesia* species responsible for human babesiosis, although, to date, most cases have been attributed to *B. microti* and *B. divergens*. These two species have been extensively studied, and in this review, we mostly focus on the antigens involved in host–parasite interactions. We present features of the major antigens, so-called Bd37 in *B. divergens* and BmSA1/GPI12 in *B. microti*, and highlight the roles of these antigens in both host cell invasion and immune response. A comparison of these antigens with the major antigens found in some other Apicomplexa species emphasizes the importance of glycosylphosphatidylinositol-anchored proteins in host–parasite relationships. GPI-anchor cleavage, which is a property of such antigens, leads to soluble and membrane-bound forms of these proteins, with potentially differential recognition by the host immune system. This mechanism is discussed as the structural basis for the protein-embedded immune escape mechanism. In conclusion, the potential consequences of such a mechanism on the management of both human and animal babesiosis is examined.

## 1. Introduction

Human babesiosis has been mainly reported in North America and Europe and has been increasingly identified in Asia [1]. On other continents, either the underestimation of diagnosis or a low risk level could explain the low rate of detected human babesiosis. As a zoonotic disease transmitted by ticks, human babesiosis results from the encounter of a parasitized vector with a susceptible host [2]. The current epidemiology of human diseases is driven by tick tropism for humans, parasites circulating in the tick population, and frequency of tick bites in shared areas (for leisure, work, or home locations). Such a situation is well illustrated by *B. divergens* human babesiosis in Europe, where a tick bite by infected *Ixodes ricinus* leads to transmission, but severe disease mainly develops in spleen-deficient patients. The *B. divergens* parasite will not pursue its development in immunocompetent humans, so human-to-human or human-to-animal transmission is considered very unlikely. In North America, severe human babesiosis caused by *B. microti* is also a great concern for immunocompromised humans, ranging from neonates to the elderly. But in the case of *B. microti*, which is able to infect immunocompetent patients, asymptomatic human carriers lead to transfusional babesiosis risk.

Some *Babesia* species have been proven to develop in human blood, but for a vast number of species, the ability to invade human red blood cells remains unknown. In addition to the well-known *B. microti* and *B. divergens*, the parasites *B. duncani* and *B. venatorum* are recognized as occasional human pathogens [3]. The latter parasites have both been recognized as relatively new *Babesia* species that can invade a human host and cause clinical diseases, but many human cases are diagnosed as babesiosis without clear identification of the parasite. A recent example described patient cases of babesiosis associated with *B. odocoilei*, a parasite closely related to *B. divergens* [4]. This report highlighted the need for an accurate diagnosis assay, efficient care of babesiosis cases, evaluation of the risk level, and adaptation of prophylaxis. Babesiosis treatment will not be the same in the case of a splenectomized patient infected by *B. divergens* compared with an immunocompetent human infected with *B. microti*, at least in terms of emergency measures. Additionally, emergence of new *Babesia* species infecting humans have to be monitored to avoid potential transfusional risk.

*Babesia* have biological features that make them unique among Apicomplexa, but they also have the same requirements as other intracellular parasites, i.e., to invade a host cell and escape the immune system. A critical step for the blood stages of *Babesia* parasites is the interval between the release of merozoites from red blood cells and successful invasion of new erythrocytes [5]. During this short period of time, merozoites are flowing in the blood stream and their surfaces are coated with erythrocyte-binding proteins exposed to immune effectors, such as antibodies. Thus, the surfaces of merozoites represent an interface between the parasite and the host cells, to which the parasite has to bind while avoiding the antibodies. Among the molecules exposed at the surfaces of merozoites, at least some glycosylphosphatidylinositol-anchored proteins (GPI-AP) can meet these contradictory requirements. These proteins are anchored at the surfaces of the merozoites, providing erythrocyte-binding sites potentially used for host cell invasion. Via mechanisms not fully determined yet, the GPI anchor allows GPI-AP to be released from the plasma membrane of the parasite. As a soluble form, the shed protein could be bound to an antibody which, then, would not interfere with the parasite. These features of GPI-AP produced by parasites highlight their critical role in successful parasite development inside the host, but also their high potential as a target for vaccine or diagnostic tools. 

Human babesiosis represents only a part of all the Apicomplexa-borne disease cases, compared with animal babesiosis (cattle, dog, etc.), malaria (humans), and many other animal or human infections. Therefore, knowledge of human babesiosis could benefit from a comparative analysis with other parasites using similar mechanisms. In this paper, we review some information about *Babesia* parasites from various hosts, focusing on GPI-AP and their dual role in invasion and immune escape. Two major antigens from the species most frequently found in humans (*B. microti* and *B. divergens*) are then presented and their features compared. These two antigens, Bd37 and BmSA1, highlight the crucial role of GPI-AP in host relationships and their high potential as a target for medical intervention. From the comparative analysis of these two proteins, critical points could be determined in order to decipher the multiple functions of such antigens. This will open discussion about the possibility of standardizing current serological assays for known human babesiosis and also speed up the process of identifying antigens of high potential for diagnosis and/or vaccination. 

## 2. Glycosylphosphatidylinositol-Anchored Proteins Are Important Antigens for Invasion and Immune Escape

In recent years, there has been significant progress in genomics and phylogeny of *Babesia*, leading to improved parasite classification [6]. Comparisons of clinical observations and biological mechanisms to study the evolutional history of each parasite will certainly help to join disparate elements into a more comprehensive picture. In particular, comparative analyses of the proteins involved in host–parasite interactions, structure determinations, and functional analyses need to be extended [7].

Briefly, focusing on GPI-anchored proteins expressed by Apicomplexa, the importance of their role in host–parasite relationships is quite well known. *Plasmodium* is outside the scope of this paper, but the example of the RTS,S vaccine highlights the role of these antigens. The RTS,S vaccine is based on the GPI-anchored circumsporozoite protein (CSP) and is currently one of the most advanced recombinant vaccines in clinical trials [8]. The GPI-anchor by itself also induces an immune response in the host, in the absence of antigenic proteins [9]. Indeed, the non-protein part of the GPI anchor remains understudied in *Babesia* despite a significant role in host–parasite interactions, as evidenced in other Apicomplexa [10,11,12]. The structure of the GPI anchor has been determined in some *Babesia* species, either experimentally or by analysis of the GPI metabolic pathway [13,14,15]. The organization of GPI-AP at the cell surface in raft or other patterns remains to be determined, as well as potential heterogeneity in the anchor bound to proteins (either glycosidic or lipidic). The fate of these proteins during parasite invasion of erythrocytes and the antigen protein shedding mechanisms also need further studies.

Some studies on *Babesia* have been driven by the need to prevent babesiosis in animals, mostly cattle or dogs [16]. The classic vaccine research scheme starts from live, attenuated, or killed parasites as antigens and progresses towards the use of recombinant molecules, either protein or nucleic acids. This process leads to the identification of various antigens that can induce protective immune responses, ranging from an avirulent parasite strain to a recombinant protein. For example, soluble parasite antigens (SPAs) have often been used as a source for crude vaccine preparations containing shed GPI-anchored proteins. In most of these efficient vaccines, the role of highly immunogenic proteins has been highlighted, and in most of the cases, the relevant proteins are GPI-anchored (Table 1). 

The results obtained from various *Babesia* species have helped to define major antigens as GPI-anchored proteins with high-level expression, high abundance in the extracellular space (culture supernatants or plasma of infected host), and high immunogenicity. Another critical feature is the function of these proteins in the host cell invasion process [17]. Interestingly, in two of the main parasites responsible for human babesiosis, such major antigens have been identified. A comparative analysis of these major antigens in the two human parasites *Babesia microti* and *Babesia divergens* (Table 2) could be an opportunity to establish a common reference and classification of antigens.

## 3. The Major Antigen in *Babesia divergens*: Bd37 Protein

*Babesia divergens*, as the main cause of bovine babesiosis in Europe and potentially fatal human infections, has been extensively studied. Research efforts towards a bovine vaccine have led to the identification of the main antigen, named Bd37, according to the molecular weight of this protein (around 37 kDa) [18]. In the first attempts to obtain an efficient vaccine, in vitro culture supernatants were used as vaccine antigens. These experiments demonstrated the high protective potential of such crude antigenic preparations [25]. A critical parameter to obtain such efficient vaccines was the inclusion of the adjuvant saponin, which was found to induce a protective response. Such a crude antigenic preparation contains both secreted antigens and shed proteins and also proteins released from lysed or dying parasites. Despite the undefined composition of in vitro culture supernatants, sometimes referred to as soluble parasite antigens (SPAs) or excreted/secreted antigens (E/S antigens), they offer the possibility for successful development of a vaccine [26]. At the same time, a similar strategy for the *Leishmania* vaccine development in dogs was also successful, leading to a commercial product [27]. Another example is the vaccine developments against canine babesiosis that have also led to a commercial product [28].

A *Babesia* vaccine based on in vitro culture supernatants, or on live parasites, requires an established long-term culture system for consistent production of antigens [29,30]. A significant drawback of this production process is the availability of a critical cell substrate, i.e., the erythrocytes. Moreover, the potential presence of an infectious element in the production process, for example, a cattle vaccine used in human food-producing animals, could be an issue in terms of product safety. Therefore, a high priority is the identification of protective components in in vitro culture supernatants, which will later be produced by biotechnological methods and safer processes.

In the case of *B. divergens*, a traditional strategy was to purify relevant molecules from in vitro supernatants [25]. Using size-exclusion chromatography, separation of crude supernatants has led to several fractions that have been tested in vaccine experiments. A fraction (called F4) containing antigens with molecular weights ranging from 17 to 50 kDa has been shown to induce a protective response in gerbils [31].

Therefore, after successful purification of native Bd37 from in vitro culture supernatants, and the characterization of this protein, some immunological detection tools have been generated, including immune serum and the so-called F4.2F8 monoclonal antibody, which was raised against the protective F4 fraction from the supernatant, hence the name of the mAb [32]. Interestingly, mAb F4.2F8 greatly reduced clinical signs. To obtain the coding sequence of the Bd37 antigen, a cDNA library from *B. divergens* merozoite mRNA was screened with immune serum [33]. Although this type of library could introduce a large bias in the representation of cell transcripts, positive clones contain sequences directly expressible in bacteria.

When Bd37 was identified, no *B. divergens* genome data were available as they were released in 2014 [34,35]. In addition, the proteomic analyses of a complex mixture with low abundance of parasite molecules among large amounts of host proteins is still highly challenging. Therefore, the systematic identification of other GPI-AP of *B. divergens* has not been published yet, although some Bd37-related genes can be found in released genomes [36,37].

**Figure 1:**
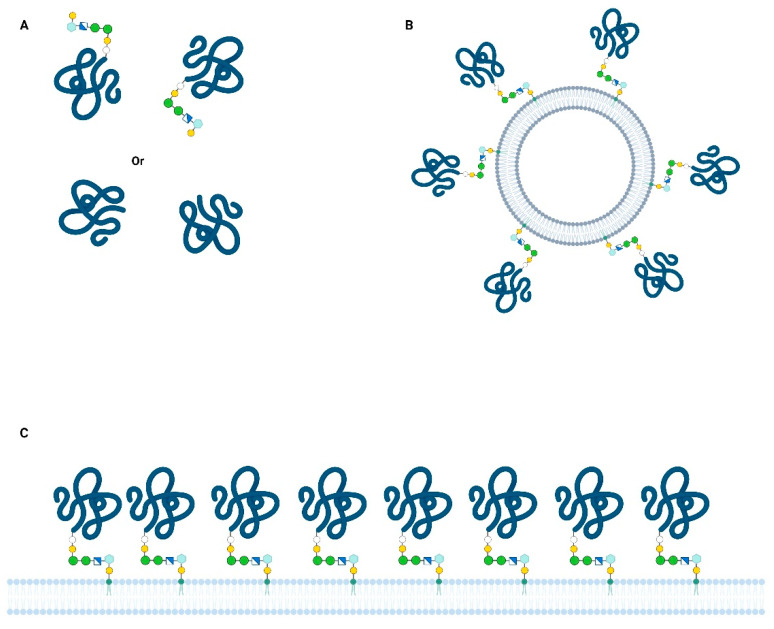
Different physical presentations of major GPI-anchored antigens in *Babesia*. Protein concentration, orientation, and degrees of freedom could influence interactions between antigens and antibodies or erythrocyte surface: (**A**) Soluble protein free in the blood and in vitro culture supernatants (e.g., Bd37 and BmSA1); shed by proteolytic or phospholipase cleavage (**B**) extracellular vesicle released by parasite, coated with GPI-anchored proteins (e.g.,: BmSA12); (**C**) GPI-anchored proteins at the plasma membrane of merozoite (either on the entire cell surface or on lipid rafts).

The production of a recombinant Bd37 protein without the use of a parasite in vitro culture was a milestone in the development of an industrial vaccine for cattle. Moreover, this recombinant vaccine induced an immune protection broader than that induced by the supernatant and was efficient against all the tested strains [38]. Nevertheless, a striking difference in recombinant vaccine antigen potency was observed, i.e., the protective immune response was only induced by a protein containing a hydrophobic peptide. Thus, such proteins associated with saponin are thought to mimic the merozoite surface and elicit an efficient immune response that is able to impair parasite development and onset of disease. In contrast, the Bd37 recombinant protein with similar immunogenicity but devoid of a hydrophobic tail induced an inefficient response, leaving parasites unaffected [39]. These results highlight a potential protein-embedded immune escape mechanism used by *B. divergens* parasites, in which Bd37 released in a soluble form (Figure 1A) acts as a shield for the Bd37-coated merozoite surface (Figure 1C).

The erythrocyte-binding function of Bd37 and the high vaccine potency of the cognate recombinant protein prompted the elucidation of the three-dimensional (3D) structure of this antigen [40]. This protein bears a protruding unstructured region in the N-terminal part of the molecule, followed by an alpha-helical domain. The structured core of the protein is organized into three subdomains stuck together by salt bridges, suggesting the possibility of conformational changes. Considering the membrane-bound form of Bd37, packed at the merozoite surface, the binding of relevant antibodies could be impaired by steric hindrance, while free soluble proteins are dispersed and fully exposed (Figure 1A,C). At the molecular level, a conformational change of Bd37 could also occur when merozoite and erythrocyte surfaces are in close vicinity, potentially inducing electrostatic bond modifications. Such a potential conformational change would expose erythrocyte-binding sites that are otherwise hidden from antibodies. Another possibility is that a conformational change leads to the displacement of bound antibodies in favor of erythrocyte receptors, making a humoral response useless for the host. Although further studies are required to totally decipher these mechanisms, the intrinsic properties of Bd37 integrate requirements for both immune escape and erythrocyte-binding functions.

## 4. The Major Antigen in *Babesia microti*: BmSA1/GPI12 Protein

A vaccine against *B. microti* for human use is not considered the best tool for human babesiosis management, in contrast to *B. divergens* and cattle babesiosis, for which vaccine research has been extensive. Therefore, in *B. microti*, research on GPI anchored antigens has been more focused on their use for detection. Nevertheless, first attempts to identify relevant antigens have rapidly led to a much more complex panel of molecules than those found in *B. divergens* [41]. Since the first genome annotation, which was published in 2012, genomic data on this parasite have been generated, including the genome analysis of several isolates [42]. These data allow better characterization of the parasite proteome and metabolic pathways, including the protein post-translational modification machinery. The immune response against *B. microti* and the most relevant antigens have been reviewed previously [43]; therefore, in this paper, we focus on one of the most characterized antigens without ignoring the role of other proteins.

BmR1_03g00785, named BmGPI12, has been identified as a GPI-anchored antigen from the *Babesia microti* R1 strain using a bioinformatics strategy based on targeting signal recognition at the N and C terminal position in amino acid sequences [44]. This strategy has been used on *B. bovis* to predict around 20 GPI-AP, a number similar to the predicted GPI-anchored proteome in *B. microti* [17]. A sequence comparison analysis indicated almost complete identity with previously identified BmSA1 in other *B. microti* strains [45]. When the human antibody response against different predicted proteins was evaluated, the high immunogenicity of this protein relative to other antigens was demonstrated [44]. In addition, strong similarities with antigens from the BMN family, which are proteins identified in the *B. microti* MN1 strain after serological screening, were observed [46]. The history and features of the BMN antigens were recently reviewed, and the name of BmBAHCS1 (*Babesia* alpha-helical cell surface) was suggested for BmR1_03g00785 [47]. The 3D structure of this antigen has not yet been experimentally determined, but sequence analysis has indicated an unstructured part, followed by an alpha-helical domain. This organization is relatively similar to the Bd37 protein, in which a disordered region is N-terminally added to a structured core of an alpha-helical protein. Another common feature shared with Bd37 is its early recognition by antibodies during the rise of an immune response [44,48].

The analysis of BmHACS1/GPI12/SA1 (henceforth referred to as BmSA1 for readability) expression in *B. microti* has revealed an unusual mechanism for releasing proteins in culture supernatants as vesicle-anchored antigens [49]. Thus, this GPI-anchored antigen has a classic cellular distribution of merozoite surface protein released into soluble form, as well as membrane-bound protein on small vesicles (Figure 1B). These vesicles appear to be loaded with some other antigens, strongly suggesting a role in host immune system manipulation. Neither soluble nor vesicle-bound BmSA1 could be involved in erythrocyte invasion without physical connection to the merozoite. These forms of the protein are produced at a high rate and therefore could be exploited as a diagnostic marker of active infection [50].

There have been some attempts to evaluate the potential of BmSA1 as a vaccine antigen and to determine the inhibitory growth potency of antibodies [51]. Showing some similarities with Bd37 in *B. divergens* vaccine experiments, the immune response elicited by recombinant BmSA1 could either be protective or with negligible effect on the infection course [52]. In some cases, antibodies against this protein could inhibit in vitro culture, suggesting blocking of the essential interaction with the host erythrocyte. The direct interaction of the recombinant protein with erythrocytes has been demonstrated, definitively making BmSA1 a major antigen of *B. microti* [19]. 

## 5. Protein-Embedded Immune Escape Mechanism: Membrane-Bound Versus Soluble Antigen

Merozoite surface proteins function as an interface between host and parasite by interacting with the immune system through effectors (antibodies, cell receptors etc.). Among these proteins exposed to the immune system are erythrocyte-binding proteins, which, in order to maintain their cell interaction ability, have limited evolutionary opportunities. This functional restraint counteracts the trend to diversify antigen sequences in response to immune pressure. Recently, the initial interaction of merozoite proteins with erythrocytes, just prior to invasion, has been documented at the cell scale in *B. divergens* [53]. This initial step seems to allow transitory interactions (quite like “touch-and-go” binding to red blood cells) or induce the wrapping of the erythrocyte membrane around the parasite, possibly triggering the invasion sequence. Such wrapping could be the consequence of a coordinated multiple low-affinity interaction between GPI-anchored merozoite proteins and erythrocyte surfaces. It is possible that such low-affinity interaction occurring at one point on the merozoite surface, possibly involving only a few proteins, can spread to other close proteins, leading to a large contact zone with erythrocytes.

The functional constraint to bind to the surface of erythrocytes limits sequence variation (polymorphism or antigenic variation mechanism) in such a protein domain, whereas their exposure to antibodies applies a selective pressure to develop escape mechanisms [7]. A common feature of both major antigens, Bd37 and BmSA1, is their ability to induce strong immune responses, although their effects against parasite development and disease onset are highly variable. The *B. divergens* vaccine model, using recombinant Bd37 with or without a hydrophobic tail, shifts the immune response from totally inefficient to a full protective immunity [39]. There is a similar situation in *B. microti*, for which different experiments have evaluated the immune response induced by various types of recombinant BmSA1 proteins and adjuvants [51,52]. In both cases, the host immune system (mostly from gerbils and mice in experimental vaccine trials) was able to raise a protective response depending on the antigen vaccine used. On one hand, the protein-embedded immune mechanism acting in GPI-anchored proteins could drive the immune system response toward soluble or vesicle-anchored antigens, almost obliterating the protective response against merozoites. On the other hand, an efficient vaccine would drive the immune response toward merozoite surface-bound proteins, either impairing the infection or at least leading to parasitemia decay and disease recovery. The balance between protective and non-protective response could then rely on the dynamics of interactions with antibodies (conformational changes) or on the local concentration of membrane-bound proteins in contrast to dispersed soluble proteins. 

In both *B. divergens* and *B. microti* species, a monoclonal antibody directed against Bd37 and BmSA1, respectively, can interfere in vivo with the target parasite. The protective effect of such monoclonal antibodies could result from the high concentration of antibody against a single epitope, or from better interaction with the membrane-bound antigen than with the soluble form. The binding of an antibody to its cognate antigen can be greatly affected by the physical state of the protein, either membrane-bound or soluble [54]. This is especially the case for GPI-anchored proteins, as these proteins could be packed in a membrane raft of potentially high density or homogeneously spread on the cell surface, which could have an impact on antibody binding (Figure 1C) [55]. For vesicle-bound antigens, it remains to be determined if the protein density and orientation are similar to the plasma membrane-bound proteins, and the interactions with antibodies would need to be characterized.

The conformational changes in Bd37 and the potential shift in functional properties of the protein, according to such a structural dynamics, are reminiscent of protein allostery, mainly described in enzymes but applicable to antigens [56]. The nature of the GPI anchor is probably the basis of the immune escape mechanism embedded in Bd37, inducing modifications in the conformational dynamics of the protein when bound to the membrane or released in soluble form. The influence of the GPI anchor on the conformational dynamics and functional properties has been shown in the prion protein, which could exist as both a membrane-bound and soluble protein [57,58]. Another level of complexity may be represented by the structural diversity of the GPI anchor. Thus, varying anchors may be attached to the protein according to the C-terminal signal processing sequence, with varying impact on protein properties [59].

In addition to protein structure, the type of GPI anchor could have an impact on the antigen presentation at the parasite surface and then affect the function of such antigen [60,61]. The genome of *B. divergens* has been released and analyzed, confirming that Bd37 is a member of a small group of related genes [36]. One of the questions is to determine the membrane pattern (localization, density, orientation, etc.) of these Bd37-related proteins and determine whether or not their GPI-anchors are identical. The diversity of the GPI-anchor structure in the different GPI-anchored proteins from parasites has not yet been extensively studied. GPI-anchor structure has an impact on protein shedding, which leads to the release of protein as soluble antigen, and potentially on the protein organization at the surface of the parasite [59,60,61]. Thus, determining, at a proteome scale, the specificity or the identity of the GPI-anchor type for each antigen will be a major milestone in the understanding of host–parasite molecular interactions.

Deciphering all the characteristics of a protective immune response is challenging, while predicting which antigen formulation will induce such protective response is even more complex. Nevertheless, there is growing evidence suggesting that mimicking the membrane-bound form of antigens instead of the soluble form is one of the keys to achieving protective immunity [62]. In the Bd37 (*B. divergens*) vaccine model, a single recombinant antigen can switch between an efficient or inefficient vaccine according to the formulation. In most of the other Apicomplexa vaccine experimental models, there is not such a great difference, in clinical signs or parasitemia, between protective and non-protective immune responses. A general trend of results in the literature indicates that a membrane-bound antigen is generally better than soluble molecules. This could be recombinant molecules, attenuated or killed whole parasites, or crude antigen preparations formulated with adjuvant. GPI-anchored Bd37 expressed on the surface of a live, heterologous recombinant vaccine (*Trypanosoma theileri*) was able to induce a protective immune response in cattle, illustrating the importance of a membrane-bound form in vaccine potency [63]. Another example is the Gp60 protein of *Cryptosporidium*, which has been expressed in *Tetrahymena* [64]. An increase in local antigen density could potentially also account for the high protection rate obtained with the R21 CSP-based vaccine [65]. These examples illustrate the importance of vaccine formulation and antigen expression to obtain efficient, protective immune responses.

## 6. Use of Major *Babesia* Antigens in the Management of Human Babesiosis: Polymorphism and Cross-Reactions

As previously mentioned, human babesiosis cases occur at the crossroad of vector, parasite, and host biology, which are evolving parameters due to climate change. Therefore, the distribution of endemic babesiosis areas will certainly change, together with the potential emergence of new zoonotic *Babesia* species. In contrast to animal babesiosis, for which a vaccine is the best control method, human babesiosis mainly needs detection tools and a risk mitigation system. Improved mapping of the high prevalence areas for parasites and vectors, together with public information, would certainly lower infection rates. In addition, rapid and reliable diagnostic assays are greatly needed both for the prevention of babesiosis (for example in blood screening for transfusion) and for patient healthcare. Acute human babesiosis requires quick parasite identification in patients before a humoral immune response can develop. A splenectomized patient with *B. divergens* babesiosis should need additional healthcare compared to an immunocompetent patient with *B. microti* babesiosis. However, there is significant interest in serological assays that could be used for epidemiological analyses.

It should be noted that the *B. microti* R1 strain was isolated in France from a patient coming from North America [41]. Therefore, people who travel and become infected with various species of *Babesia* may need an appropriate diagnostic test far from the source of the infection area [66]. This supports the development of a broad diagnostic system that is able to detect as many different species as possible [67]. Molecular tools could detect almost all *Babesia* species, depending on the oligonucleotides used in a PCR, but serological assays are much more challenging. In addition to the number of species to be included, the polymorphism of each antigen could impair the detection of antibodies raised against distantly related parasites [37]. Therefore, an ideal “universal” serological assay should probably use different recombinant proteins, covering most species and strains encountered in patients, and be delivered in a multiplexed format. A panel of recombinant proteins could advantageously be included in a *Babesia* antigen toolbox, which would be useful for the development of serodiagnostic assays, but also for basic research on protein structure determination and functional analysis.

According to the results obtained with Bd37 and BmSA1 in two *Babesia* species, achieving a broad, multiplexed, serological assay might not be as difficult as expected. In addition to classic immunofluorescence assays using native antigens from in vitro cultures or infected animals, recombinant proteins have been successfully used in serological assays [68,69]. Some initial studies on *B. divergens* antigens have compared the immune response raised in different hosts and demonstrated a similar profile of recognized proteins [48]. Thus, a standardized serological assay could be efficiently developed in parallel for both cattle and humans, using a common panel of recombinant proteins, therefore reducing costs. Moreover, despite the polymorphism found on Bd37, the recombinant protein based on the Rouen 1987 sequence is able to bind antibodies elicited against various strains, suggesting that a few antigens, or possibly only one, would allow the achievement of a broad-spectrum serological assay for *B. divergens*. In the case of *B. microti*, the BmSA1 antigen seems to detect antibodies against many different strains [45].

Other species can cause human babesiosis, for example *B. duncani* and *B. venatorum*, but also new emerging species, for which major antigens have not yet been characterized or even identified. Extrapolating results from other *Babesia* species (Table 1), there is a high probability of finding relevant diagnostic antigens for a serological assay among the GPI-anchored proteome of these parasites [15]. Achieving an efficient recombinant vaccine is not the main objective for managing human babesiosis; efforts should focus on the main antibody targets with high expression levels that are most favorable for diagnostic assays. In vitro cultivation of *B. duncani* will ensure enough biological material for genome, transcriptome, and proteome analyses, as well as in-cell experiments [70]. Concerning *B. microti*, a long-term in vitro cultivation system is not available yet, as for many other species. However, the identification of a major antigen and quickly including it in a serodiagnostic toolbox, in the case of a new emerging species, will remain highly challenging. Progress in genome sequencing methods and annotation pipelines would help researchers avoid the in vitro cultivation bottleneck and allow them to generate data from limited samples.

## 7. Conclusions

Major antigens in *Babesia*, such as erythrocyte-binding proteins with high immunogenicity, have mostly been found in the GPI-anchored proteome. The Bd37 protein was identified in *B. divergens* using a classic and time-consuming approach based on in vitro cultivation of the parasite, but genome analysis and recombinant protein expression have speeded up the process, as described for the BmSA1 protein from *B. microti*. These antigens, expressed as recombinant proteins, could form the basis of high-performance serodiagnostic assays.

These two antigens highlight the role of GPI-anchored proteins in *Babesia* biology and the complexity of their functional relationships with the host. Protein structure and dynamics, proteome organization, carbohydrate heterogeneity and GPI-anchor diversity, polymorphism, and antibody cross-reactivity need to be further explored to decipher such complex interactions. The first step of erythrocyte invasion relies on GPI-anchored parasite proteins, and in addition to major antigens, there are many other molecules with still unknown roles. Achieving a complete atlas of GPI-anchored proteins, including quantitative expression data, cellular and membrane organization, mechanisms and rates of soluble protein release, and protein and anchor structure, should be an important milestone. The knowledge about these proteins can then be applied to their usage in diagnostic or therapeutic applications as vaccines.

The immune response against these major antigens, at cell and antibody level, also deserves further studies. In particular, the definition of correlates of protection is needed to differentiate a protective from a non-protective response. The identification of biomarkers associated to immune protection is a prerequisite to the development of a predictive assay for the protection status of a subject, either human or animal. As an example, current IFAT or ELISA serological assays can detect antibodies against intraerythrocytic merozoites or recombinant proteins in sera from protected but also unprotected animals without evidencing differences between them. Considering that neutralization assays (growth-inhibitory assays) are not satisfying and need rather large amounts of the sample, the development of differentiating immune status assays for *Babesia* has high interest and could then be extrapolated to other Apicomplexa parasites. 

There is a need to keep the basic science effort on *Babesia* at a high level, in particular, in a climate change context. The complete antigenic landscape, displayed by a GPI-anchored proteome in membrane-bound or soluble state, and the role of major antigens in this immune interface are still not fully understood. This knowledge is nevertheless critical to select and properly express recombinant antigens that could lead to efficient vaccines. Although well-established models have to be maintained to allow in-depth experiments, it should be anticipated that new parasite species could emerge, and then methods should be set up to ensure quick and efficient management of these human babesiosis cases. Currently, the generation of large data sets (annotated genome, transcriptome etc.) for a new species requires the propagation of the parasite to obtain sufficient amounts of biological material (DNA, RNA etc). Otherwise, GPI proteome prediction strategy based on hydrophobic signal recognition (which does not need sequence homologies) can be applied to many pathogens [71]. Until now, such data cannot easily be obtained from clinical human samples; therefore, animal inoculation continues to be used to isolate parasites. Although not needed for routine analysis, it should be performed in the case of an unknown parasite or atypical babesiosis, even if current animal models probably do not reflect the entire range of human susceptibility to *Babesia* species. 

For serological analysis, whatever the commercial outcome, a panel of recombinant antigens should be the most efficient tools to cover the broad range of human *Babesia* parasites. Such an antigen toolbox will grow with each new protein or parasite described and should allow the definition of a common framework for serological assay development. The current markets of diagnostic assays for human and animal babesiosis are not the same, but it will be interesting to discuss a common toolbox extracted from scientific knowledge, based on a comparison between *Babesia* species. As discussed in this paper, major antigens and cognate antibodies could be used for vaccines, various types of serological assays, and antigen capture assays. This could be used to define biomarkers of protection, biomarkers of exposition, or biomarkers of active infection, which will be useful for human and animal babesiosis management. 

## Figures and Tables

**Table 1:** Some examples of major antigens found in *Babesia* sp. These proteins are all GPI-anchored at the surface of merozoites and are released into host blood or in vitro culture supernatants (++: evidence of high potential, +/−: no clear evidence or no data supporting high potential).

*Babesia* Species	Protein	Expression Level	Diagnostic Potential	Vaccine Potential	Ref.
*B. divergens*	Bd37	++	++	++	[18]
*B. microti*	BmSA1/GPI12	++	++	++	[19]
*B. bovis*	MSA1	++	++	++	[20]
*B. bigemina*	Gp45	++	++	++	[21]
*B. canis*	Bc28	++	++	+/−	[22]
*B. canis*	BcMSA/CBA	+/−	+/−	++	[23,24]

**Table 2:** Comparison between Bd37 and BmSA1 features (+: effective activity, −: no activity).

	Bd37	BmGPI12/BmSA1
Related genes in genome	2 to 6 genes (need further analysis)	BMN family (>15 members) Not all are GPI-anchored
Protein global structure	mainly α-helical protein (2jo7)	BAHCS domain (α-helical)
GPI anchor core structure	Man2-GlcN	Man2-GlcN
GPI lipid moiety	palmitate	not determined
Erythrocyte binding activity	+	+
Growth-inhibitory antibodies	−	+
Early detected by antibodies	+	+
In vivo protection	effective or not	Effective or not
Secreted form	soluble	vesicle-bound and soluble

## Data Availability

Not applicable.
